# Supplementary material for: Early assessment of fungal and oomycete pathogens in greenhouse irrigation water using Oxford nanopore amplicon sequencing
Source: PLoS One. 2024 Mar 15;19(3):e0300381. doi: 10.1371/journal.pone.0300381 (PMC10942031; doi:10.1371/journal.pone.0300381)
Supplement: S1 File — (PDF) [file pone.0300381.s001.pdf]

Supplementary file

Early assessment of fungal and oomycete pathogens in greenhouse irrigation water using Oxford nanopore amplicon sequencing

Enoch Narh Kudjordjie[1], Anne Saaby Schmidt-Høier[2], Mai-Britt Brøndum[2], Mads Grønvald Johnsen[2], Mogens Nicolaisen[1], Mette Vestergård[1]

Additional materials and methods

Greenhouse water purification and DNA extraction

The frozen water samples were thawed and filtered using 0.22 µm Sterivex™ sterile filters. Filtering was done manually by filling 50 ml syringes with water, fitting the filters onto the adapter and pressing the plunger gently until all the water is filtered out. This step was repeated until all the required water volume (250 ml) for a sample is filtered and the ends of the sterivex filter is capped. DNA extraction from the Sterivex™ sterile filters was done using the DNeasy® PowerWater® Sterivex™ extraction kit, by following the manufacturer's instructions. Briefly, Sterivex filters were placed vertically (with the inlet cap upward) and 900 µL of ST1B buffer was added, then recapped and mixed in a vortex with a horizontal adapter with the inlets facing out at a minimum speed for 5 minutes. This was repeated by rotating the filters in 90 degrees and vortexed. 900 µL of solution MBL was carefully added and the filter units positioned vertically with the inlet upward and incubated at 90 ˚C for 5 minutes in a water bath. The filters were cooled at room temperature with caps tightened and vortexed for 5 minutes by placing them horizontally, with the inlet facing out to a vortex adapter. The lysate is transferred from the filters to a 3 mL syringe and then into a into a 5 mL Powerbeat tube, followed by horizontal vortexing for 5 minutes at maximum speed and centrifuging at 4000 x g for 1 minute. The lysate is then carefully transferred into clean 2.2 mL collection tubes, and 1.6 µL of RNAs (100 mg/mL) is added, incubated at 37 ˚C for 30 minutes in a water bath. 300 µL IRS solution is added to the tube and vortexed and then incubated at 4 ˚C 5 minutes. 3 ml of Solution MR was added to the collection tube containing supernatant and vortexed to mix. The MB Spin Columns are placed in a 2.2 ml collection tube and 700 µl of supernatant added, followed by centrifugation for 1 min at 13,000 x g. The filtrate was discarded and repeated until all the supernatants had been loaded into

the MB Spin Column. This step was repeated about 7 times for each processed sample, followed by washing with 0.7 ml of Solution PW, and then centrifuging for 1 min at 13,000 x g. The flow-through was discarded and the content was washed with 0.7 ml of ethanol and centrifuged for 1 min at 13,000 x g. The MB Spin Column were then removed and placed in a 2.2 ml collection tube, centrifuged at 13,000 x g for 2 min to completely dry the membrane. Finally, to elute the DNA, the MB Spin Columns are transferred to a new 2.2 ml collection tube and add 100 µl of Solution EB to the center of the white filter membrane, followed by spinning down at 13,000 x g for 1 min at room temperature. The extracted DNA was quantified using Qubit 3 Fluorometer and stored DNA at -20 C.

Artificial Spike-ins

Forward primer binding site highlighted in green
Reverse primer binding site highlighted in yellow
Length between primers(amplified regions)highlighted in red

1. **Mirrored from MT453296.1 Fusarium oxysporum isolate DSM100409_FOX (1135 bp. Rv region 563) (538 bp)**

TTACTGGTTCTGAAGGAGGTATGCTGAAAACTCTGGAACAAAGTGGTTGCGGAGGCGACCCC
ATTACTAGAACATTTGAGCCAAACCCTCACAAGTGTCCTGTTCACCATGCGGCTCCGTGCCC
GACTAGAATGGCCCTCGGCAGGGCAAGAGACCGCCCAAATCCCCAGTCTTTGTCTCTCAATG
TATAAAATGAGTCTATAAATACCATCAAAACTAAGCAACAACTTGGTTCTCTAGCTACGGTC
TTCAAGAAGTAGTAAAACGACGATGAATAGCGGTTAAGTGTAGACTTAAGACGCTACTAAGT
AAGTTTCTAACGTTACACGCTGACCGCCCGGGCGGTCTTATGTCCGTACGACTGCGAGCTTC
CCAACTTTCGACACGAACGGTTGTGGTTTTGCGCTCAGTTGCGCTTAAGTTAAACTCCCTGG
CGGTTATCGAGCTGCATGCGATACCTAAAATGATGACATTGCTCCCTGCTAATGGTGCACCG
GCGCCCAAATTGCCGTCTTCAACCCCAGTTGTAAGGATAGGCTCCATAAGGATTTCAAGTCG
CACTATACGAAGAGGCGAATACAAAGAAAAGTAGGGACAACATGATCCCGTGTGAGCGGCAC
AACGGCGAATTAAACTCGAGGTCTAAAGTCCGGGCTCTCAATGTTGAGCGGAGATGTTTAGT
TTTCATATCCGTGGCGTCCTTGAGCCTGGGCAAGGTCAGATACCGCACGAGAGTGGGTGGTC
TGCCCAACCGTAGGTAATGTCTCTAACTTCCTTGAGAGCTGAGCAAGGGTTTGATTCTCGTC
GTAGGAGGGTAAATTCTGTATATAATCGAAATCACCGGCCATAATAGCCAGAGTGAACACGC
GGCTAGTGAGAAAAGTAGAAAGATTTCACGATTGAGAGAAAGCATGAAAAATTGTTAAAGTG
AAGGGAAAGCAGTATTTGCGGGTTCAGACTAATTGGTTCTGGGGTCTACTGACCCCTCTCCT
TTTCACGCCGGACCTGATTGACTACGAGGGGCCCCTTGCGGAAATAGTGTAAGGGCGCTTCT
CTCGGTTGTGAGGGGCCACCCGATACATAATGTGAGGGGGGGTCGCTTGGAGTCACGTCTAC
GCGGTCGTAGGAAATGC

2. **Mirrored from MK649847.1 Saccharomyces cerevisiae strain S430b (885 bp. NB: 44TTs were removed (799 bp)**

TTACTGGTTCTGAAGGAGATATGCTGAAAACGGAACAAAGTGGATGCGAAGGCGTCCAAATT
ACTAGTAATTTAAAGAAAGTTAATAGGTATTGTTTTGAGAACGGTTTCGAGAGTACCGGGTC
ATTTAACAGAAGAAGAGGGTAGAGGGCCGACCTGTTCGCGTCGGGCGCGTGAAGGATCGTTC

TTTGAATGTTCCGTTCTTTCTCAAACCTTACTAGAGAGTGGTTCGTGTCTTGGATATTGTTA
AAATTAACATAACTTTGCCTCACACAACATTTTGAGGTGGTTTCTATACCTTCAACTTACGG
GTTTGCTAACGAGCGAGACCCGGGACAAACAATGTTTAACAAACACTTATCTATAAATTACA
AAAACTGGCTTTTAAGAAAAGGTCAATATAAAATTCAAAAATTGGCAACAACTTGGTTCTCT
AGCTACGCTCTGCAAGAAGTAGTAAAGCGACAATGCATAGCCGTTAAGTGTGTCCTTAAGAA
GCTACTAAGCAAGTTTCTAGCGTTACACGATGGTTCCCCGGGGGACCTTTTGTCCGTACTAC
TGCGAGTACTCTTCCTTTCTCTTACAAAGTGATGGTTTCTCATAGTGAATTGAGGTTGTTAA
AGTTCTTCCGGTCTGTAGGTTACCCTTGGAGAGAAATGCGTCTCTTATGGAGTTCGTGAACG
TAATTGCTGGCACATTGGATCGGCGTCAACTTCTAATGAGTCATATTAAGGTTATGCTAGCT
ATTCGGAGAGAAGAAGCGGATCTGCCTTGTAACAAGTTTGAAATTCTAAACTCCATGAGGAT
GGAAAGTCGCCCAATACGAATTCGCGAATAACTAGGAG

3. Mirrored from MW366735.1 Pythium sp. isolate 11_1 (865 bp) (809 bp)

GTGGATGGGAAGGCGTCCAAATTACTAGGAAAACCACACCCCTTTCAAAAGCCAAGTGCATA
TCAATGTTTCGTGTCTTGAGGCTCTTCGAAGTCGAGAGGGGTGGAAGCTTAATTCGCTCCGT
CTGATGTTTCATGTAGTTACCCAAATCATAATCAAACATATCAAGTGCAAAAGCCTGTTTCT
GAAAACTAATTTTGTTCAACAATAGGTGACGACTCGGATCTGTAGCTACACGCTGCAAGAAG
TAGTCAAGCGTCAATGCATAGCCGTTAAGCGTTGACTTAAGAAGCTACTGAGCAAGTTTTAA
ACGTTACACGTTGGGCTTTCAAGGTCCGTATGTCCGTATGCCTGTGACTAAAACTACATGCT
TTCCGTTCGTGTTTTTTAAGAACTGATCGGTAGAGAGGAGTGTCAGATCGCTCTGTGTCTC
CCTCAGAGAGGAGGCTTGAGCGCAGATAAATTTCCCCGCTTGCATGTGTTCTTTCTGTAGAA
TTTGTCAGTGTGAAACGCAAGCTTTTGTCTAGTGTTCGCTAGGTGTGAGCTCGTGCTTCAGC
GGTACAGGATTGCGAAGGAAATTTATCTCCAATGTATGGCGGGGCTTCGGATGTTTCAGCCC
GTCAGTCGACTTTTGTTGAGCTTTCTTGTCTGTGGAGTTCGTTCTGTCCAAACGGAGTGTCG
GGTCTGGTTATTGGTAAAGATGATGTGTGTCGTTGAAGGCGAATTCTCCTTCGATCTCATGA
GATTGGAACGGAGGAAGTTTAACCACTGCTGATAGGACTATTTAGTTCTCGCGGTAGGTTAA
TCTTCTATAGTCCATCAGAACGGAAAGTCGCCCAATACGAATTCGCGAATAACTTGGTG

4. Mirrored from EF126351.1 *Phytophthora infestans* isolate UASWS0289 (893 bp) (829 bp)

GGATGCCTTTGCGTCCAAGTTACTAGGAAGTCCACACCATCTTTCAAAAACAAGTGCACCCC
AACTTTGCGGTTGATAACTCATTCTGGGCGGCGGCGGTTTTCGGTCGTGCGGTCGTTAGTCG
TCATCGTCCCGAGCGGCGGAAAACTAGGTTTGCGAGTCTGGCTTCAGATGATCGAGTTATTT
TTTCTTCCCAAATTCATAATTCATTCATATTAGTAGCAGGGGTGCGTCTCTGAAGATCAATT
TTTTCAACGATAGGTGACGACTCGGATCTGTAGCTACACGCTGCAAGAAGTAGTCAAGCGTC
AATGCATAGCCGTTAAGCGTTGACTTAGGAAGCTACTGAGCAAGTTTTAAACGTTATACGTT
GGGCCTTCAAGGTCCTGATGTCCGTATGCCTGTGACTAAAACAACATGCTTTCGGTTCTGCC
TTCCTTGTGGCTGATGCGTAGAGGAGAAGTGTAGACGCGTTCTGTGCTTTTGGTTGGTCAGC
CAGGTTTCCTGAGCTCATGTAAATTCATGTCAAATCGTTTCTCTGGTGAAAACCTCGTTACG
GTCAGGTGTTGGGTTATCGTCGGTTGAGCGATTTGGCCAGCGCGTCGTCTGTGTAATTGCGG
TCGTAAAGAGGGTGCTTAGCCGGTTGGTATAAGTCGGCTTTTCGCGTAACTAGTGGGTTAGT

CGTCCTTTGTAGTGCGGTAAGTGGTCAGTCTCGGTACCCGGTTCGATTGCTAAGTTTATGTCGTTTCGGATGAAGCGTTCGGCGGTGAGCCGTCGGCTCTAGCTGGGAAAGGGTTTATATGTGTTGTAGTATGGCTTCTTAACTCTACATAGTCCAGGAGAACGGACTGTCGCCCATTACGAATTCAAGAATAACTATAGTAGGAGGCGGG

Processing of MinIon sequencing files

General description:

All data is generated with Oxford Nanopore MinION by AU Flakkebjerg. Fast5 files were basecalled using guppy, high accuracy model(available from ONT).
Sequences are demultiplexed with guppy and adapters removed using porechop. Sequences are then filtered on quality and length using filtlong. Finally sequences are mapped against the UNITE reference database using minimap2 and are then summarised to operational taxonomic units (OTUs) in R.
OTUs are visualised with heatmaps showing the relative amount of a given OTU for each sample.

LAB 1:
fast5 data generated by AU Flakkebjerg
Includes: Both fungi and oomycetes
Date of sequencing: 2021-09-30
TI folder name: 2021_11_01_AU

Programs used in the analysis
guppy: version 6.1.2+e0556ff, high accuracy model
porechop: --check-reads 1000
filtlong: min. length: 200, max. length: 5000, min. accuracy (based on q-score): 90
minimap2: version: 2.22-r1101, settings: -ax map-ont --secondary=no
samtools:version 1.11 (using htslib 1.11)
R: version 4.2.0 (2022-04-22) -- "Vigorous Calisthenics"

Reference database used for mapping: UNITE v. 8.2 all eukaryotes, including global and 97% singletons.
Spike sequences were manually added to the reference before mapping
Barcodes used: 1-28

LAB 2 (oomycetes):
fast5 data generated by AU Flakkebjerg
Includes: oomycetes
Date of sequencing: 2022-01-12
TI folder name: 2022_01_24_AU_OomyArtSpikein2Programs used in the analysis
guppy: version 6.1.2+e0556ff, high accuracy model
porechop: --check-reads 1000
filtlong: min. length: 200, max. length: 5000, min. accuracy (based on q-score): 90
minimap2: version: 2.22-r1101, settings: -ax map-ont --secondary=no
samtools:version 1.11 (using htslib 1.11)
R: version 4.2.0 (2022-04-22) -- "Vigorous Calisthenics"

Reference database used for mapping: UNITE v. 8.2 all eukaryotes, including global and 97% singletons.
Spike sequences were manually added to the reference before mapping
Barcodes used: 1-60

LAB 2 (fungi):
fast5 data generated by AU Flakkebjerg
Includes: fungi
Date of sequencing: 2022-02-17
TI folder name: 2022_03_17_AU_FungiArtSpikeIn2

Programs used in the analysis
guppy: version 6.1.2+e0556ff, high accuracy model
porechop: --check-reads 1000
filtlong: min. length: 200, max. length: 5000, min. accuracy (based on q-score): 90
minimap2: version: 2.22-r1101, settings: -ax map-ont --secondary=no
samtools:version 1.11 (using htslib 1.11)
R: version 4.2.0 (2022-04-22) -- "Vigorous Calisthenics"
Reference database used for mapping: UNITE v. 8.2 all eukaryotes, including global and 97% singletons.
Spike sequences were manually added to the reference before mapping
Barcodes used: 31-90

LAB 3:
Demultiplexed fastq data generated by AU Flakkebjerg
Includes: oomycetes
Date of sequencing: 2022-04-01
TI folder name: 2022_04_22_AU_OomySpikeIn_Bckgd_seq
Programs used in the analysis
filtlong: min. length: 200, max. length: 5000, min. accuracy (based on q-score): 90
minimap2: version 2.17-r941, settings: -ax map-ont --secondary=no
samtools: version 1.11 (using htslib 1.11)
R: version 4.0.4 (2021-02-05) -- "Lost Library Book"

Reference database used for mapping: UNITE v. 8.2 all eukaryotes, including global and 97% singletons.
Spike sequences were manually added to the reference before mapping
Barcodes used 1-72

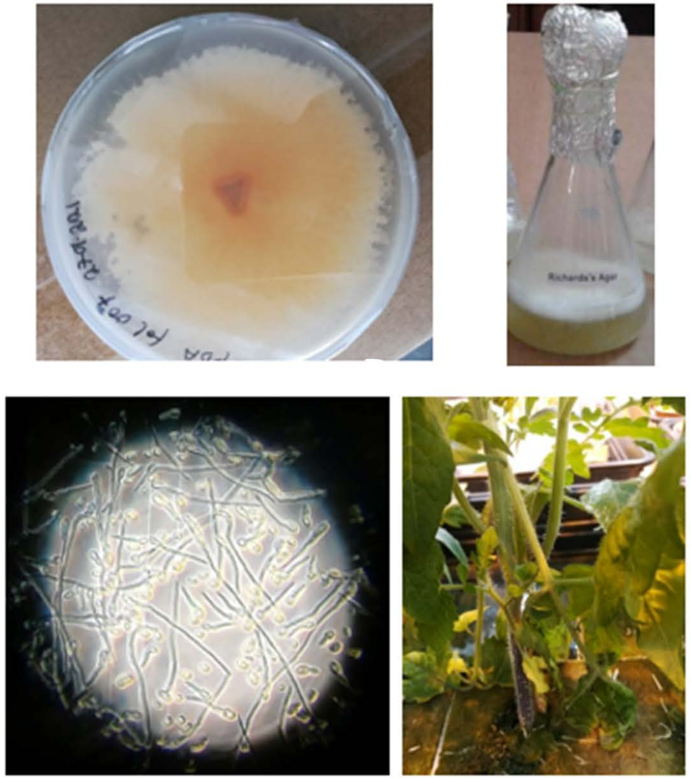

Figure S1. *Fusarium oxysporum* spore purification: A) *Fusarium* growth on PDA plate B) *Fusarium* broth for spore production C) *Fusarium* mycelia and spores viewed under the microscope and D) wilt symptoms on tomato after 6 weeks of infection.

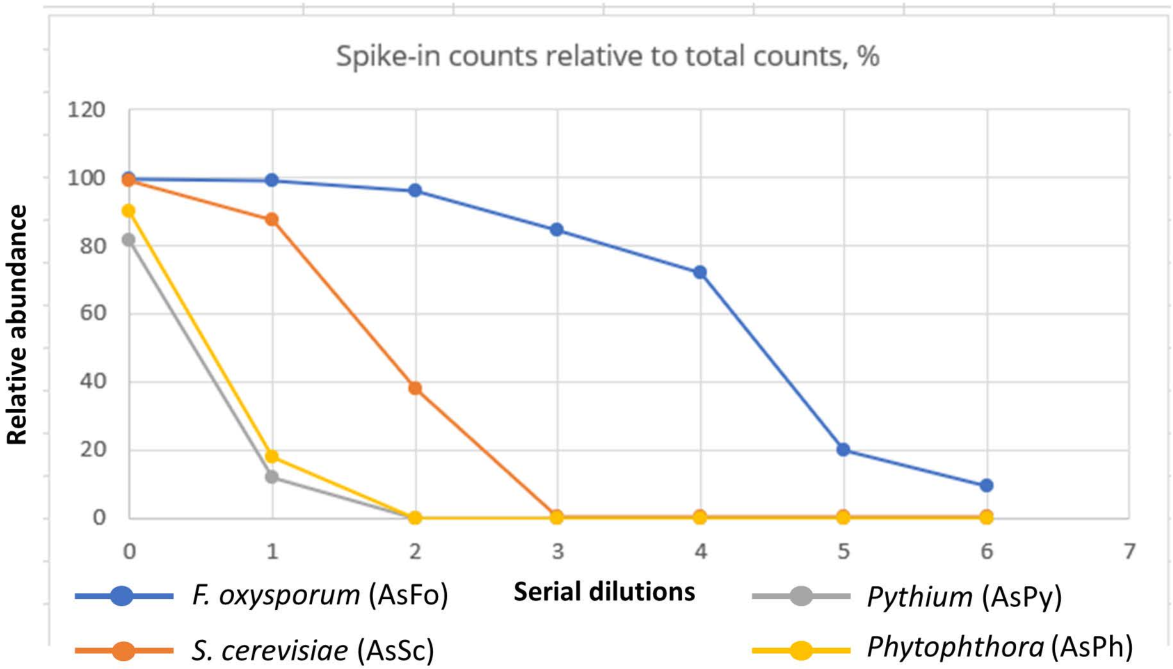

Figure S2. Plot showing spike-in counts relative to the percent total count in samples spiked serially (6-folds) with fungal and oomycetes artificial spike-ins

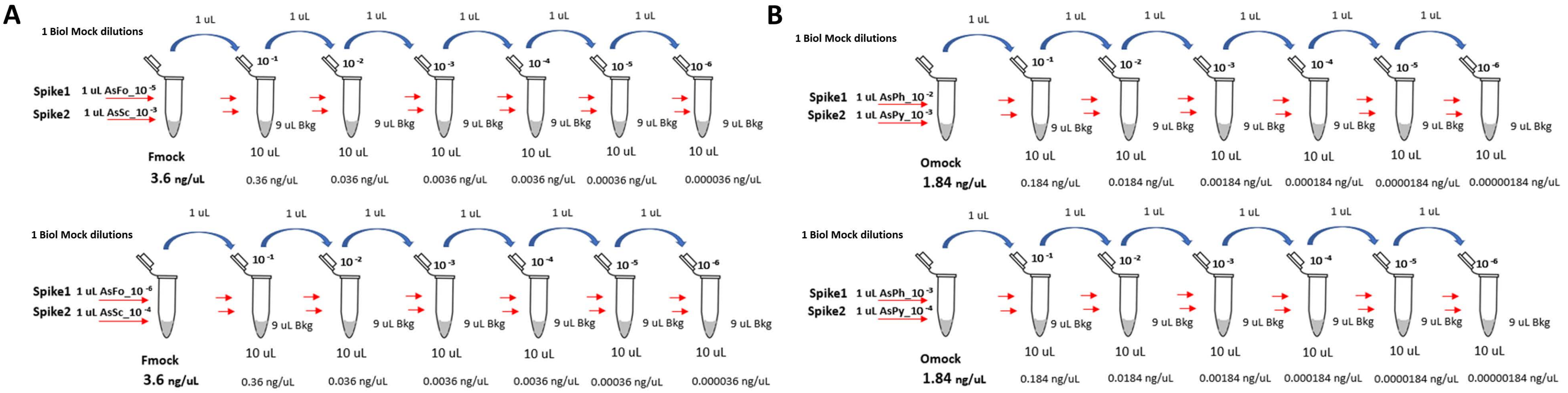

Figure S3. Mock dilution and spiking of A) fungal and B) oomycete artificial spike-ins scheme. Fmock and Omock were serially diluted (6 × 10-fold) into a background greenhouse water DNA sample with a DNA concentration of 1.56 ng/ul (Figure S3A, B). We constructed two Fmocks with artificial spike-ins at two different dilutions (AsFmock1= [AsFo:10^{-5} and AsSc: 10^{-3}] and (AsFmock2= [AsFo: 10^{-6} and AsSc: 10^{-4}]) and two Omocks (AsOmock1= [AsPh: 10^{-2} and AsPy: 10^{-3}] and (AsOmock2= AsPh: 10^{-3} and AsPy: 10^{-4}]) 1 uL of the selected spike-in sample was added to each dilution of the mock samples.

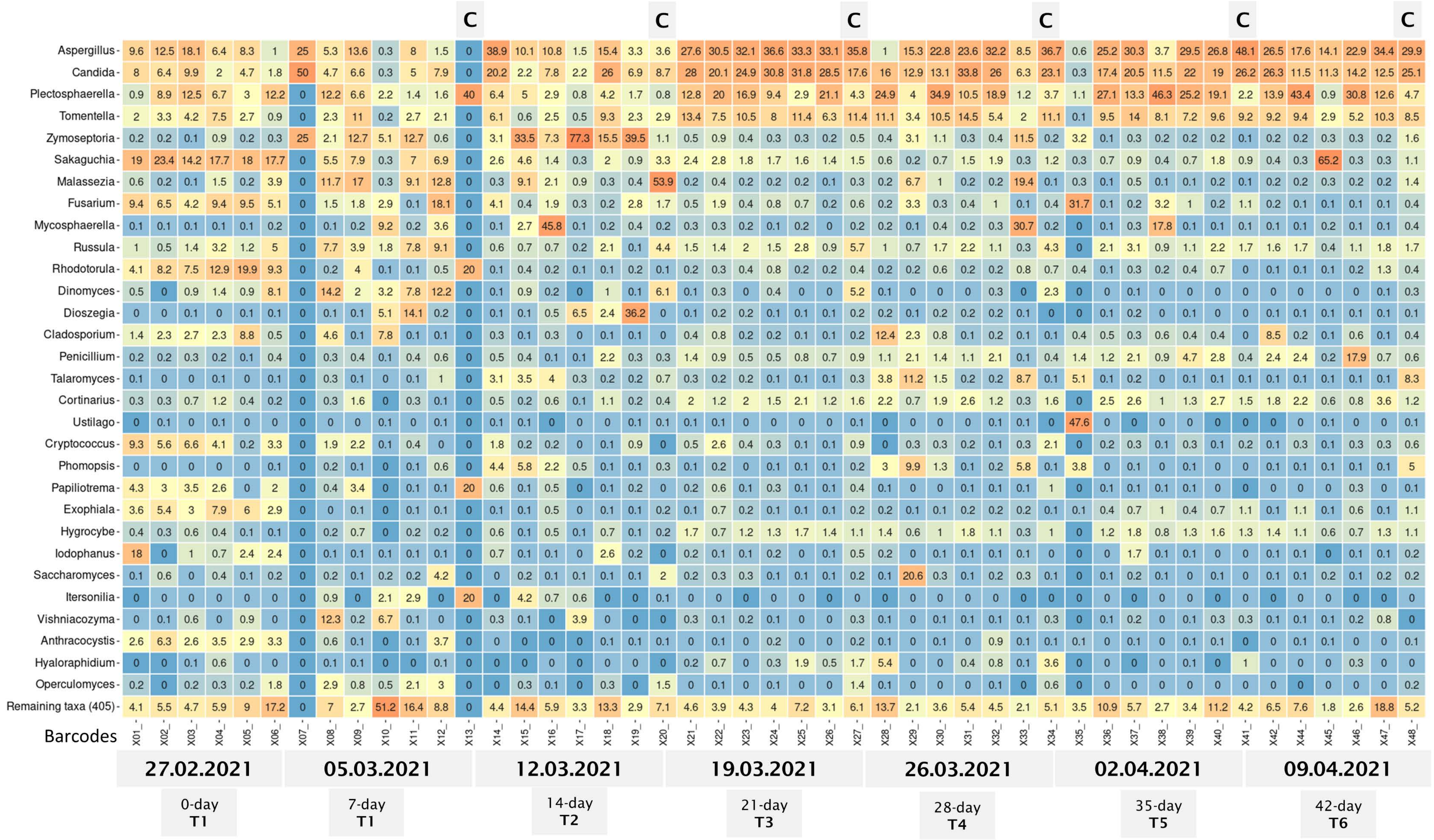

Figure S4. Heatmap of fungal relative abundances in water samples inoculated with *Fusarium* and control (C) across different sampling time points. Taxonomic classification at genus level is shown in the y-axes, while samples represented by barcode number are indicated in the x-axis.

Table S1: **Details of greenhouse water sampled in 2020**

Sample Number	Quantity	Filtered_date	Extracted_date	Collection point	Plant	Plan stage	Symptoms	Qubit (ng/uL)
1	500 mL	07.09.20	13.09.21	drain	Cucumber	matured	non detected	1.36
2	500 mL	07.09.20	13.09.21	drain	Cucumber	matured	non detected	0.812
3	500 mL	09.09.20	13.09.21	drain	Cucumber	matured	non detected	11.5
4	200 mL	10.09.20	13.09.21	drain	Tomato	matured	non detected	0.364
5	200 mL	10.09.20	13.09.21	drain	Tomato	matured	non detected	1.01
6	200 mL	10.09.20	13.09.21	drain	Tomato	matured	non detected	0.246
7	200 mL	10.09.20	16.09.21	drain	Tomato	matured	non detected	Too low
8	200 mL	14.09.20	16.09.21	drain	Tomato	matured	non detected	0.642
9	200 mL	14.09.20	16.09.21	drain	Tomato	matured	non detected	Too low
10	200 mL	14.09.20	16.09.21	drain	Tomato	matured	non detected	0.216
11	200 mL	14.09.20	16.09.21	drain	Tomato	matured	non detected	Too low
12	200 mL	14.09.20	16.09.21	drain	Tomato	matured	non detected	0.278
14	200 mL	14.09.20	16.09.21	drain	*Aeschynanthus*	young	non detected	3.94
15	200 mL	14.09.20	16.09.21	drain	*Aeschynanthus*	young	non detected	0.408
16	200 mL	15.09.20	16.09.21	drain	*Aeschynanthus*	young	weak	0.272
17	200 mL	14.09.20	16.09.21	drain	*Aeschynanthus*	young	weak	Too low
18	200 mL	14.09.20	16.09.21	drain	*Aeschynanthus*	young	weak	Too low
19	200 mL	14.09.20	16.09.21	drain	*Aeschynanthus*	matured	weak	0.232
20	200 mL	14.09.20	17.09.21	drain	*Aeschynanthus*	matured	weak	0.308
21	200 mL	14.09.20	17.09.21	drain	*Aeschynanthus*	matured	weak	6.74
22	200 mL	15.09.20	17.09.21	drain	*Aeschynanthus*	matured	weak	1.34
23	200 mL	15.09.20	17.09.21	drain	*Aeschynanthus*	matured	weak	0.688
24	200 mL	15.09.20	17.09.21	drain	*Aeschynanthus*	matured	weak	1.84
25	200 mL	15.09.20	17.09.21	drain	*Aeschynanthus*	matured	weak	0.612
26	200 mL	15.09.20	17.09.21	drain	*Aeschynanthus*	young	weak	4.08
27	200 mL	15.09.20	17.09.21	drain	*Aeschynanthus*	matured	weak	1.61
28	200 mL	15.09.20	17.09.21	drain	*Aeschynanthus*	matured	weak	2.52
29	200 mL	15.09.20	17.09.21	drain	*Aeschynanthus*	matured	weak	1.04
30	200 mL	15.09.20	17.09.21	drain	*Aeschynanthus*	young	weak	4.38

Table S2: Biological mock composition and concentrations

Sample	Isolate	Amount pooled (uL)	Volume ratio	Mock concentration ng/uL
FMock	*Fusarium oxysporum*	5	1	1.75
	Saccharomyces cerevisiae	5	1	
	Verticillium dahliae	5	1	
OMock	*Phytophthora infestans*	5	1	0.84
	P. fragariae	5	1	
	Globisporangium intermedium	5	1	

Table S3. Absolute read counts obtained from MinIon sequencing of samples spiked with fungal and oomycetes artificial spike-ins

Barcode number	Total number of reads	Spike in type	Number of reads for spike in (only available for some)		
1	239791	AsSc	237240		
2	109992	AsSc	96355		
3	37775	AsSc	14396		
4	25404				
5	9762				
6	29510				
7	17357				
8	269880	AsFo	269127		
9	189920	AsFo	187777		
10	165980	AsFo	159778		
11	102869	AsFo	87139		
12	27391	AsFo	19715		
13	23186	AsFo	4623		
14	20240				
15	119767	AsPh	107675		
16	85966				
17	79499				
18	135048				
19	147179				
20	123042				
21	118758				
22	85326	AsPy	69423		
23	69778				
24	103867				
25	78959				
26	83704				
27	81515				
28	92874				

Table S4: Summary of sequences obtained for green house water 2020 (GHW2020) samples and barcodes after quality filtering using ONT MinIon sequencing.

Sample	Sample ID	Target	Amp conc (ng/uL)	#Read	#Barcode	Sample ID	Target	Amp conc (ng/uL)	#Read	#Barcode
01	01	Fungi	7	818	1	30	Oomycetes	21.8	15178	30
02	02	Fungi	6.54	146	2	31	Oomycetes	20.6	7802	31
03	03	Fungi	5.16	351	3	32	Oomycetes	26	4356	32
04	04	Fungi	10	393	4	33	Oomycetes	13.8	2380	33
05	05	Fungi	21.9	741	5	34	Oomycetes	20.7	10024	34
06	06	Fungi	8.23	800	6	35	Oomycetes	18.1	5798	35
07	07	Fungi	5.31	2	7	36	Oomycetes	13.3	2973	36
08	08	Fungi	19.1	452	8	37	Oomycetes	14.9	5059	37
09	09	Fungi	5.68	4	9	38	Oomycetes	13.7	5435	38
10	10	Fungi	13.8	855	10	39	Oomycetes	14.9	3699	39
11	11	Fungi	4.42	0	11	40	Oomycetes	14.2	1145	40
12	12	Fungi	4.79	5	12	41	Oomycetes	12.1	2745	41
14	13	Fungi	21	2025	13	42	Oomycetes	36.8	11387	42
15	14	Fungi	11.7	1089	14	43	Oomycetes	23.5	6347	43
16	15	Fungi	10.9	213	15	44	Oomycetes	15	5267	44
17	16	Fungi	5.95	358	16	45	Oomycetes	13.7	1340	45
18	17	Fungi	6.18	119	17	46	Oomycetes	13	3376	46
19	18	Fungi	8.33	1740	18	47	Oomycetes	16.9	3702	47
20	19	Fungi	13.3	38	19	48	Oomycetes	15	2753	48
21	20	Fungi	31.9	167	20	49	Oomycetes	37.8	3208	49
22	21	Fungi	29.4	259	21	50	Oomycetes	36.7	27693	50
23	22	Fungi	29.6	334	22	51	Oomycetes	29.2	24138	51
24	23	Fungi	52	128	23	52	Oomycetes	33.7	10225	52
25	24	Fungi	14.1	611	24	53	Oomycetes	22.9	6592	53
26	25	Fungi	18.4	1318	25	54	Oomycetes	30.1	4403	54
27	26	Fungi	23.7	4882	26	55	Oomycetes	23.9	7461	55
28	27	Fungi	22.3	410	27	56	Oomycetes	24.7	17298	56
29	28	Fungi	15.3	1773	28	57	Oomycetes	23.9	10076	57
30	29	Fungi	37.4	440	29	58	Oomycetes	24.7	4642	58

Table S5: Summary of sequences obtained from infection model experiment samples and barcodes after quality filtering using ONT MinIon sequencing

Sample	Sample ID	Target	Amp conc (ng/uL)	#Read	#Barcode
01	01	Fungi	24.2	6214	01
02	02	Fungi	13.7	5408	02
03	03	Fungi	20.7	26644	03
04	04	Fungi	21.5	18250	04
05	05	Fungi	13.2	5456	05
06	06	Fungi	18.5	18960	06
07	07	Fungi	4.28	4	07
08	08	Fungi	20.8	3762	08
09	09	Fungi	19	2776	09
10	10	Fungi	13.1	3182	10
11	11	Fungi	13.3	4359	11
12	12	Fungi	11.5	2203	12
13	13	Fungi	4.28	5	13
14	14	Fungi	26.9	2995	14
15	15	Fungi	14.1	1508	15
16	16	Fungi	17	3327	16
17	17	Fungi	27.3	4075	17

18	18	Fungi	19.7	14887	18
19	19	Fungi	15.4	3104	19
20	20	Fungi	17.6	2442	20
21	21	Fungi	46.7	5294	21
22	22	Fungi	23	14637	22
23	23	Fungi	50	6059	23
24	24	Fungi	too high	18481	24
25	25	Fungi	55	8193	25
26	26	Fungi	too high	10551	26
27	27	Fungi	36.5	17634	27
28	28	Fungi	9.21	1240	28
29	29	Fungi	8.97	10520	29
30	30	Fungi	29.2	16193	30
31	31	Fungi	37.7	7635	31
32	32	Fungi	22	23362	32
33	33	Fungi	8.06	1530	33
34	34	Fungi	40	3614	34
35	35	Fungi	8.02	720	35
36	36	Fungi	38.4	40603	36
37	37	Fungi	35.2	5918	37
38	38	Fungi	35.2	17892	38
39	39	Fungi	51	10404	39
40	40	Fungi	34.8	6097	40
41	41	Fungi	24.3	7488	41
42	42	Fungi	60	9357	42
44	44	Fungi	43.9	29762	44
45	45	Fungi	5.6	6127	45
46	46	Fungi	23.2	9130	46
47	47	Fungi	18.2	6664	47
48	48	Fungi	20.7	3384	48

Table S6: Summary of sequences obtained from artificial spike-ins assessment run (Lab 1) samples and barcodes after quality filtering using ONT MinIon sequencing

Sample	Target	#Read	#Barcode	Amp conc (ng/uL)
01	Fungi	297186	1	16.2
02	Fungi	139879	2	8.56
03	Fungi	54798	3	6.41
04	Fungi	38558	4	6.53
05	Fungi	18860	5	7.41
06	Fungi	47579	6	8.45
07	Fungi	26844	7	7.49
08	Fungi	371304	8	33
09	Fungi	267731	9	33.7
10	Fungi	224285	10	21.8
11	Fungi	141910	11	11.2
12	Fungi	40605	12	10
13	Fungi	36160	13	6.88
14	Fungi	32705	14	9.88
15	Oomycetes	160823	15	9.53
16	Oomycetes	112163	16	8.6
17	Oomycetes	100800	17	8.44
18	Oomycetes	179998	18	10.1
19	Oomycetes	188312	19	8.12
20	Oomycetes	182012	20	7.85
21	Oomycetes	154688	21	7.94
22	Oomycetes	108131	22	11
23	Oomycetes	89529	23	6.41
24	Oomycetes	144388	24	12.3
25	Oomycetes	105319	25	11.8

26	Oomycetes	108467	26	9.59
27	Oomycetes	105928	27	9.15
28	Oomycetes	130290	28	8.24

Table S7: Summary of sequences obtained from biological mock and artificial spike-ins assessment run (Lab 2) samples and barcodes after quality filtering using ONT MinIon sequencing.

Sample	Sample ID	Target	Amp conc (ng/uL)	#Read	#Barcode	Sample ID	Target	Amp conc (ng/uL)	#Read	#Barcode
31	31	Fungi	47.6	104	31	1	Oomycetes	44.7	350	1
32	32	Fungi	43.3	545	32	2	Oomycetes	31.8	8539	2
33	33	Fungi	38	1580	33	3	Oomycetes	23.6	20469	3
34	34	Fungi	37.8	2436	34	4	Oomycetes	20	19245	4
35	35	Fungi	38.3	3007	35	5	Oomycetes	19.8	23692	5
36	36	Fungi	36.2	2934	36	6	Oomycetes	19.6	42374	6
37	37	Fungi	51	88	37	7	Oomycetes	44.1	178	7
38	38	Fungi	42.6	348	38	8	Oomycetes	33.9	3567	8
39	39	Fungi	42.3	746	39	9	Oomycetes	23.1	14208	9
40	40	Fungi	39.9	5427	40	10	Oomycetes	22.3	30450	10
41	41	Fungi	36.1	4852	41	11	Oomycetes	20.9	40058	11
42	42	Fungi	38.5	2026	42	12	Oomycetes	15.4	32485	12
43	43	Fungi	48.3	69	43	13	Oomycetes	43.4	300	13
44	44	Fungi	45.7	318	44	14	Oomycetes	31.6	3081	14
45	45	Fungi	39.7	1405	45	15	Oomycetes	22	28303	15
46	46	Fungi	36.1	1342	46	16	Oomycetes	17.1	43693	16
47	47	Fungi	36	1790	47	17	Oomycetes	18.6	50245	17
48	48	Fungi	37.3	1721	48	18	Oomycetes	15.5	49560	18
49	49	Fungi	49.2	78	49	19	Oomycetes	44.7	372	19
50	50	Fungi	40.7	758	50	20	Oomycetes	34.7	6642	20
51	51	Fungi	38	1077	51	21	Oomycetes	23.6	33011	21
52	52	Fungi	37.3	1462	52	22	Oomycetes	17.9	42036	22
53	53	Fungi	37.6	2139	53	23	Oomycetes	17.3	44418	23
54	54	Fungi	36.8	4850	54	24	Oomycetes	16.7	46689	24
55	55	Fungi	52	72	55	25	Oomycetes	42.1	374	25
56	56	Fungi	45.7	368	56	26	Oomycetes	32.5	3343	26
57	57	Fungi	40.1	1186	57	27	Oomycetes	23.2	25410	27
58	58	Fungi	33.3	1748	58	28	Oomycetes	18	42342	28
59	59	Fungi	34.9	2345	59	29	Oomycetes	17.7	46469	29
60	60	Fungi	34.7	923	60	30	Oomycetes	18.4	42641	30
61	61	Fungi	47.7	99	61	31	Oomycetes	44.6	470	31
62	62	Fungi	47.2	128	62	32	Oomycetes	32.2	4694	32
63	63	Fungi	43	1707	63	33	Oomycetes	21.1	22669	33
64	64	Fungi	40.1	3801	64	34	Oomycetes	16.7	42189	34
65	65	Fungi	38.4	3512	65	35	Oomycetes	18.1	37078	35
66	66	Fungi	40.2	3847	66	36	Oomycetes	17.7	36859	36
67	67	Fungi	59	6	67	37	Oomycetes	49.5	69	37
68	68	Fungi	55	173	68	38	Oomycetes	41.8	473	38
69	69	Fungi	48.9	540	69	39	Oomycetes	30.9	3797	39
70	70	Fungi	42.7	1150	70	40	Oomycetes	21.9	19331	40
71	71	Fungi	44.3	2622	71	41	Oomycetes	18.5	30388	41
72	72	Fungi	46.9	4379	72	42	Oomycetes	19.8	44836	42
73	73	Fungi	37.2	2550	73	43	Oomycetes	18.6	60581	43
74	74	Fungi	59	8	74	44	Oomycetes	49.3	78	44
75	75	Fungi	53	151	75	45	Oomycetes	40.4	377	45
76	76	Fungi	41.5	766	76	46	Oomycetes	32.5	3310	46
77	77	Fungi	39.9	3787	77	47	Oomycetes	23	18919	47
78	78	Fungi	38.5	4777	78	48	Oomycetes	17	50203	48
79	79	Fungi	38.8	2378	79	49	Oomycetes	18	56132	49
80	80	Fungi	38.1	897	80	50	Oomycetes	18.9	50509	50
81	81	Fungi	59	13	81	51	Oomycetes	48.8	38	51
82	82	Fungi	53	212	82	52	Oomycetes	43.3	353	52
83	83	Fungi	49.3	907	83	53	Oomycetes	35	3496	53
84	84	Fungi	46	1166	84	54	Oomycetes	23.1	39531	54
85	85	Fungi	41.8	4395	85	55	Oomycetes	25.1	57106	55
86	86	Fungi	41.2	2853	86	56	Oomycetes	16.4	56191	56

87	87	Fungi	39.5	2130	87	57	Oomycetes	17.1	45206	57
88	88	Fungi	41.2	3514	88	58	Oomycetes	18.4	50412	58
89	89	Fungi	39.5	3657	89	59	Oomycetes	16.8	26872	59
90	90	Fungi	42.7	4758	90	60	Oomycetes	16.6	40285	60

Table S8: Summary of sequences obtained when assessing the effects of background sample concentration on oomycetes artificial spike-ins assessment run (Lab 3) samples and barcodes after quality filtering using ONT MinIon sequencing.

Sample	Sample ID	Target	Amp conc (ng/uL)	#Read	Barcode #
1	1	Oomycetes	12.6	11433	1
2	2	Oomycetes	16.6	6525	2
3	3	Oomycetes	18.1	11093	3
4	4	Oomycetes	13.1	4517	4
5	5	Oomycetes	15.2	6540	5
6	6	Oomycetes	17.1	5542	6
7	7	Oomycetes	18.1	8425	7
8	8	Oomycetes	15.8	5726	8
9	9	Oomycetes	16.3	12866	9
10	10	Oomycetes	14.9	9126	10
11	11	Oomycetes	16.7	10308	11
12	12	Oomycetes	16.9	13280	12
13	13	Oomycetes	14.9	11244	13
14	14	Oomycetes	15.1	9085	14
15	15	Oomycetes	15.9	5706	15
16	16	Oomycetes	14.1	8015	16
17	17	Oomycetes	14.5	7438	17
18	18	Oomycetes	15.7	10246	18
19	19	Oomycetes	15.7	10479	19
20	20	Oomycetes	16.1	11452	20
21	21	Oomycetes	17.2	3026	21
22	22	Oomycetes	15.7	6432	22
23	23	Oomycetes	15.8	7443	23
24	24	Oomycetes	17.1	11644	24
25	25	Oomycetes	14.9	12798	25
26	26	Oomycetes	21	11824	26
27	27	Oomycetes	22.9	15723	27
28	28	Oomycetes	19.8	18928	28
29	29	Oomycetes	19.8	12907	29
30	30	Oomycetes	23	17004	30
31	31	Oomycetes	21.9	16647	31
32	32	Oomycetes	23.1	17136	32
33	33	Oomycetes	23.5	16353	33
34	34	Oomycetes	20.2	14665	34
35	35	Oomycetes	21.2	15007	35
36	36	Oomycetes	21.8	19463	36
37	37	Oomycetes	22.6	15945	37
38	38	Oomycetes	24.3	16267	38
39	39	Oomycetes	23.3	9309	39
40	40	Oomycetes	23.1	16434	40
41	41	Oomycetes	22.8	18769	41
42	42	Oomycetes	19.7	17871	42
43	43	Oomycetes	21.7	19508	43
44	44	Oomycetes	20.2	16285	44
45	45	Oomycetes	22.4	15636	45

46	46	Oomycetes	21.4	15586	46
47	47	Oomycetes	23.8	10614	47
48	48	Oomycetes	22.5	16361	48
49	49	Oomycetes	24.4	13612	49
50	50	Oomycetes	24.4	13516	50
51	51	Oomycetes	24.2	14448	51
52	52	Oomycetes	25.1	19342	52
53	53	Oomycetes	25.5	11948	53
54	54	Oomycetes	25.3	13526	54
55	55	Oomycetes	23.9	17612	55
56	56	Oomycetes	24.3	17470	56
57	57	Oomycetes	25.2	10189	57
58	58	Oomycetes	22.9	11256	58
59	59	Oomycetes	24.7	19730	59
60	60	Oomycetes	24.9	15930	60
61	61	Oomycetes	25.7	14299	61
62	62	Oomycetes	22.8	10810	62
63	63	Oomycetes	24.9	24827	63
64	64	Oomycetes	23.9	15925	64
65	65	Oomycetes	24.1	25746	65
66	66	Oomycetes	25.8	17130	66
67	67	Oomycetes	25.1	19863	67
68	68	Oomycetes	24.3	13984	68
69	69	Oomycetes	26.4	21450	69
70	70	Oomycetes	25.7	15062	70
71	71	Oomycetes	26.6	12741	71
72	72	Oomycetes	25.8	16467	72
