# Supplementary material for: Early assessment of fungal and oomycete pathogens in greenhouse irrigation water using Oxford nanopore amplicon sequencing
Source: PLoS One. 2024 Mar 15;19(3):e0300381. doi: 10.1371/journal.pone.0300381 (PMC10942031; doi:10.1371/journal.pone.0300381)
Supplement: Attachment — Submitted filename: Response to Reviewers.docx [file pone.0300381.s002.docx]

**Response to reviewers comment**

When submitting your revision, we need you to address these additional requirements.

1. Please ensure that your manuscript meets PLOS ONE's style requirements, including those for file naming. The PLOS ONE style templates can be found at 
<https://journals.plos.org/plosone/s/file?id=wjVg/PLOSOne_formatting_sample_main_body.pdf> and 
<https://journals.plos.org/plosone/s/file?id=ba62/PLOSOne_formatting_sample_title_authors_affiliations.pdf>

2. We note that the grant information you provided in the ‘Funding Information’ and ‘Financial Disclosure’ sections do not match. When you resubmit, please ensure that you provide the correct grant numbers for the awards you received for your study in the ‘Funding Information’ section.

3. Thank you for stating the following financial disclosure: 
"GUDP (project no. 32002)" 
Please state what role the funders took in the study.  If the funders had no role, please state: ""The funders had no role in study design, data collection and analysis, decision to publish, or preparation of the manuscript."" 
If this statement is not correct you must amend it as needed. 
Please include this amended Role of Funder statement in your cover letter; we will change the online submission form on your behalf.

4. Please expand the acronym “GUDP” (as indicated in your financial disclosure) so that it states the name of your funders in full.
This information should be included in your cover letter; we will change the online submission form on your behalf.

5. Thank you for stating the following in the Acknowledgments Section of your manuscript: "The research was funded by GUDP (project no. 32002)."
We note that you have provided funding information that is not currently declared in your Funding Statement. However, funding information should not appear in the Acknowledgments section or other areas of your manuscript. We will only publish funding information present in the Funding Statement section of the online submission form. 
Please remove any funding-related text from the manuscript and let us know how you would like to update your Funding Statement. Currently, your Funding Statement reads as follows: "GUDP (project no. 32002)"

Please include your amended statements within your cover letter; we will change the online submission form on your behalf.

6. In the online submission form, you indicated that Data presented in this study are available upon request.
All PLOS journals now require all data underlying the findings described in their manuscript to be freely available to other researchers, either 1. In a public repository, 2. Within the manuscript itself, or 3. Uploaded as supplementary information.
This policy applies to all data except where public deposition would breach compliance with the protocol approved by your research ethics board. If your data cannot be made publicly available for ethical or legal reasons (e.g., public availability would compromise patient privacy), please explain your reasons on resubmission and your exemption request will be escalated for approval.

The raw sequence files have been deposited in NCBI SRA under the bioproject # PRJNA1063628.

7. Please include captions for your Supporting Information files at the end of your manuscript, and update any in-text citations to match accordingly. Please see our Supporting Information guidelines for more information: <http://journals.plos.org/plosone/s/supporting-information>.

We have included captions of the Supporting Information in the manuscript file.

Additional Editor Comments :
Please specifically note the comments by Reviewer #2 in your revision, and update taxonomic names as appropriate.


[Note: HTML markup is below. Please do not edit.]

Reviewers' comments:

Reviewer's Responses to Questions

**Comments to the Author**

1. Is the manuscript technically sound, and do the data support the conclusions?

The manuscript must describe a technically sound piece of scientific research with data that supports the conclusions. Experiments must have been conducted rigorously, with appropriate controls, replication, and sample sizes. The conclusions must be drawn appropriately based on the data presented.

Reviewer #1: Yes

Reviewer #2: Yes

2. Has the statistical analysis been performed appropriately and rigorously?

Reviewer #1: Yes

Reviewer #2: Yes

3. Have the authors made all data underlying the findings in their manuscript fully available?

The [PLOS Data policy](http://www.plosone.org/static/policies.action#sharing) requires authors to make all data underlying the findings described in their manuscript fully available without restriction, with rare exception (please refer to the Data Availability Statement in the manuscript PDF file). The data should be provided as part of the manuscript or its supporting information, or deposited to a public repository. For example, in addition to summary statistics, the data points behind means, medians and variance measures should be available. If there are restrictions on publicly sharing data—e.g. participant privacy or use of data from a third party—those must be specified.

Reviewer #1: No

Reviewer #2: Yes

4. Is the manuscript presented in an intelligible fashion and written in standard English?

PLOS ONE does not copyedit accepted manuscripts, so the language in submitted articles must be clear, correct, and unambiguous. Any typographical or grammatical errors should be corrected at revision, so please note any specific errors here.

Reviewer #1: Yes

Reviewer #2: Yes

5. **Review Comments to the Author**

Please use the space provided to explain your answers to the questions above. You may also include additional comments for the author, including concerns about dual publication, research ethics, or publication ethics. (Please upload your review as an attachment if it exceeds 20,000 characters)

Reviewer #1: The manuscript reads very well and I had an easy time getting through it. I just have a few minor comments for authors (see below).

Page 6 and 7 under Data processing and analysis, I suggest authors cite filling, porechop, guppy, minimap2, or at least provide a link to where software could be downloaded.
We have provided links to all packages as suggested.

Page 9. Add the species, so consider changing "...the oomycete Phytophthora (accession number:
EF126351.1) and Pythium (accession number: MW366735.1)..." To "...the oomycete Phytophthora infestans (accession number: EF126351.1) and Pythium sp. (accession number: MW366735.1)"
We have added the species name as suggested.

Pythium intermedium was transferred to Globisporangium intermedium. I suggest authors use the new name (see Uzuhashi et al. 2010 "Phylogeny of the genus Pythium and description of new genera" and Nguyen et al. 2022 "Whole genome sequencing and phylogenomic analysis show support for the splitting of genus Pythium" We thank the reviewer for this update. We have changed the name from *Pythium intermedium* to *Globisporangium intermedium.*

I suggest italicizing the genus names in the figures, if not too much of a hassle.

We have italized the genus names as suggested.

I suggest authors deposit raw data generated into a repository like NCBI SRA, and state the accession number in the manuscript.

The raw sequence files have been deposited in NCBI SRA under the bioproject # PRJNA1063628.

Reviewer #2: This manuscript presents early assessment potential of used of MinION amplicon sequencing for environmental monitoring in irrigation water. They use spike material in irrigation water and evaluate the tool for identification of Fungi and Oomycete pathogens. I think this is valuable work and information that is need to evaluate the tools, however I think they can go more far in the analysis of it and state more about the finding of non spike material. They evaluate some potential but not too clear what the best dilution or way to do it more far, they did not made the analysis to the species level but only to the genera level.
Specific comments:

Abstract: I think they can add more information in abstract about type of samples, numbers more details on results. Region of amplicon used...

We have edited the abstract as suggested.

P3L69, should rewrite this sentence, a verb I think is missing.

Response

We did not detect any mistakes

P5 Greenhouse samples and material should be presented in a table to get more clear.

We have provided a table to give an overview as suggested in the supplementary table S1.

L150 need # for the supplement info in.

We have revised the text.

L157 any info about the DNA conc. used.

We have provided the DNA concentrations in the supplementary Table S4.

L204, info on Miracloth.

It is a common autoclavable material used in microbiology for filtration of e.g. spore suspensions.

L246, please check new litterature about Pythium intermedium, now Globisporangium and could be a complex species.

We thank the reviewer for this update. We have changed the name from Pythium intermedium to *Globisporangium intermedium*.

L258, the spike-in preparation not always to clear to follow, the plasmid and not plasmid prep ...

We have added a detailed reference for the plasmid preparation. The described method used follows the protocol by Blackburn et al., 2019. Doi: http://dx.doi.org/10.1038/s41596-019-0175-1.

L299 Concentration of DNA, not always clear?

We have provided the DNA concentrations of all samples used in each experiment for MinIon sequencing in the supplementary Table S2 and S4.

L304-305 on water or plasmid material, the 1.56ng/ul?

The DNA concentration of the water was 1.56ng/ul. The plasmid mocks were serially diluted, thus they have respective serial dilution concentrations (Supplementary Table S4).

L325, seems results and discussion are put together in what I seen in <https://journals.plos.org/plosone/s/submission-guidelines> seems they are supposed to be distinct sections?

We had several experiments, where new experiments were included to address questions raised by the preceding experiments. We believe that the merged results and discussion section best reflects this flow of reasoning and makes reading much easier.

L376 and Figure 2 are very low correlation.

Fusarium reads significantly decline with increasing time in both Tray1 and tray 2, while the control tray was expectedly non-significant.

L410- seem you only have genera information, why and it is possible to get to the species level, would have been great information on the use of the method and limit. I believe information need to be add and also part of your limitation in your bioinformatic pipeline, should be part of discussion.

The main challenge of fungal species level resolution is the incompleteness of reference databases limiting the accurate and precise identification (species level) of fungal taxa, as previously described Ohta et al. 2023 doi: <https://doi.org/10.1038/s41598-023-37016-0>). We have added this limitation to the text in the manuscript.

Figure 3B do we see Phytophthora?

Phytophthora was detected in very low abundance (< 0.01). Only taxa with relative abundance of > 0.01 are shown in Figure 3B.

L449 I believe you want to say Phytophthora fragaria and not Pythium intermedium here in parenthesis.

We thank the reviewer for his oversight. We have corrected the name to *P. fragariae* in the text.

L461 more info about the amplification bias and/or references.

The observation described in lines 469-479 led us to speculate an amplification bias as previously reported by Chen et al., 2016 (referenced accordingly).

L461, interesting but I believe more work need to be done in analysis, info about the species level, any other info from the water samples, important species find, concern any info about the other genera found, any concern, any problem they could cause...Return from L347... Peronospora...

Zooming-in into fungal species is challenging, primarily due to the quality of current databases as mentioned in my earlier response. However, we were able to profile the targeted species and spike-ins used in the study using the MinIon sequencing.

Detailed information about the water samples used are provided in supplementary Table S1.

Regarding concerns of other species, we address particularly the high abundance of Sellaphora and Cyclidium in Lines 367-368.

Figure 1 the names of plant hosts and Fungi and Oomycete do not necessary align with the samples position? Recheck it.

We thank the reviewer for pointing out. We have edited figure 1 by aligning sample names, positions to fungi and oomycetes categories.

Recommendation on concentration what type of spiking, info sequences of water without spike material only?

Although we used relatively few spikes and it is thus difficult to draw firm conclusions, we observed that spikes with shorter lengths gave reads in most dilutions compared with the longer spikes. Therefore, we recommend short-length spikes. We also observed that the background concentration affects spike-in performance. Future studies are therefore needed to test several artificial spikes for benchmarking quantitative MinION sequencing.
